# Functional Analysis of a Cotton TPX2-like Gene, GbTPX2-35, in Regulating Fiber Cell Development and Strength in *Gossypium barbadense*

**DOI:** 10.3390/genes17040395

**Published:** 2026-03-30

**Authors:** Yajie Duan, Qianqian Han, Ruihong Zeng, Yongsheng Cai, Xiaowei Niu, Yuhong Wen, Xiaoju Liu

**Affiliations:** 1 College of Ecological Landscape Engineering, Xinjiang Agricultural Vocational and Technical University, Changji 831100, China; dyj547525782@126.com (Y.D.); hanqq71@163.com (Q.H.); z741119789@163.com (R.Z.); 2 College of Agronomy, Xinjiang Agricultural University, Urumqi 830052, China; cys0620@126.com (Y.C.); niuxiaowei2000@163.com (X.N.); 17677420397@163.com (Y.W.)

**Keywords:** *TPX2*, *WDL2*, microtubule, cotton

## Abstract

Background/Objectives: Among cotton species, *Gossypium barbadense* produces the strongest fibers. Examining cytoskeletal dynamics in single epidermal cells of *G. barbadense* ovules offers a direct approach to investigating fiber quality. Microtubules are major cytoskeletal components whose organization and dynamics are precisely regulated by microtubule-associated proteins (MAPs). However, information on the TPX2 family remains limited, and characterizing its features in *G. barbadense* is critical to clarifying the role of TPX2 family members in fiber strength formation. Methods: Using the *Arabidopsis thaliana* TPX2 sequence as a reference, 40, 49, 26, and 26 TPX2 family members were identified in the genomes of *G. barbadense*, *Gossypium hirsutum*, *Gossypium arboreum*, and *Gossypium raimondii*, respectively. We further analyzed the expression pattern of *GbTPX2-35* and validated its function via virus-induced gene silencing (VIGS). Results: In *G. barbadense*, *GbTPX2-35* (*Gbar_D11G59825.1*) was significantly upregulated in fiber samples of the parental lines at 25 days post-anthesis, and this expression pattern was further validated in *G. barbadense* lines with extreme fiber strength phenotypes. Next, VIGS-mediated silencing of *GbTPX2-35* downregulated the transcript levels of cellulose synthase and microtubule-related protein genes, a finding further validated by mature fiber strength phenotypic data. Conclusions: This study preliminarily validated a pathway in which *GbTPX2-35* regulates fiber strength by coordinating cellulose biosynthesis with microtubule cytoskeleton dynamics, providing valuable candidate genes and theoretical support for molecular breeding of high-strength cotton fibers.

## 1. Introduction

*Gossypium barbadense* is renowned for its outstanding fiber length and strength, and deciphering the mechanisms underlying its superior quality is a current research focus [1]. Previous studies have completed large-scale expressed sequence tag (EST) analysis during the fiber development period of *G. barbadense*, laying the foundation for investigating genes specifically expressed in its fibers [2]. Through the study of genetic mapping and analysis of gene expression characteristics, candidate genes preferentially expressed during fiber development have been identified [3]. These studies collectively reveal that the fiber quality traits of *G. barbadense* involve a complex genetic regulatory network. Cotton fibers are single-celled trichomes derived from the ovule epidermis [4]. The morphogenesis of cotton fibers highly relies on the cytoskeleton, particularly the dynamic reorganization of the microtubule system [5,6]. Numerous studies have confirmed that microtubules (MTs) and microtubule-associated proteins (MAPs) work together to maintain cytoskeletal dynamics and the arrangement of cellulose microfibrils, among other functions [7,8,9]. The cotton microtubule-associated protein GhMAP20L5 mediates fiber elongation by interacting with the tubulin protein GhTUB13 [10]. Another microtubule-associated protein, GhCLASP2, has been identified and verified to play an important role in cotton fiber development [11]. These findings further confirm that microtubule-associated proteins directly influence the morphogenesis of fiber cells by regulating microtubule dynamics.

TPX2 is a conserved microtubule-associated protein that was first identified in Xenopus egg extracts and is broadly recognized as essential for spindle assembly in animals [12,13]. TPX2 homologs occur across taxa, including plants [14]. Plant TPX2 proteins contain an Aurora-binding domain, a TPX2 import domain (PF12214), and a TPX2 domain (PF06886) [15]. The TPX2 family has numerous members involved in regulating various plant developmental processes [16,17,18]. Overexpression of *ATWVD2* leads to right-handed helical twisting in *Arabidopsis thaliana* roots and hypocotyls, accompanied by defects in cell elongation and abnormal microtubule alignment [19]. Another member of the TPX2 family, AT*WDL3*, regulates light-dependent hypocotyl elongation via the ubiquitin-26S proteasome-dependent pathway; compared to the wild type, its overexpression lines exhibit shorter hypocotyl cells, while RNA interference lines show longer hypocotyl cells [20]. *WDL5* stabilizes microtubule structures and regulates the arrangement and stability of cortical microtubules in *A. thaliana* [21]. In Populus trichocarpa, 19 TPX2 family members have been identified, which are involved in regulating development, cell elongation, and microtubule organization [22]. In Eucalyptus grandis, a TPXL gene family comprising twelve members has been identified, and its overexpression causes the right-handed twisting of cotyledon petioles [23]. *LcWDL1*, a member of the TPX2 family, is speculated to enhance lentil yield by participating in microtubule organization and cell expansion [24]. All these studies indicate that when TPX2 family members are regulated, corresponding structural abnormalities in microtubule alignment occur.

Further research has found that the arrangement direction of microtubules directly determines the deposition direction of fiber microfibrils. Cortical microtubules can regulate the transverse arrangement of microfibrils by guiding the movement of cellulose synthase complexes (CSCs) along specific directions, which is crucial for maintaining the crystallinity and mechanical properties of the cell wall [9]. Using visualization techniques, it can be directly demonstrated that CSCs are functionally associated with microtubules, and their movement trajectories are guided by microtubules [25]. After ethylene treatment, the direction of microtubules in pea epicotyl cells changes, simultaneously affecting the arrangement of cellulose microfibrils [26]. Based on the above research, it is indicated that multiple microtubule-associated protein genes, including the TPX family genes, play important roles in the dynamic changes in the cytoskeleton and the arrangement and deposition of fiber microfibrils. However, existing studies have only focused on *Gossypium hirsutum*, where *GhWDL3* inhibits fiber cell elongation during key stages of fiber development [27]; while *GhWDLA7* may affect microtubule structure by interacting with *GhTUA2*, thereby participating in the cell wall biosynthesis process and ultimately regulating cotton fiber synthesis [28]. This has led to an insufficient understanding of the molecular basis for the formation of superior fiber traits in sea island cotton, particularly regarding the mechanisms of action of microtubule-associated proteins such as the TPX2 family, constituting a research gap in this field.

Collectively, the direct or indirect roles of TPX2 family members in plant development highlight the value of investigating this family in cotton, particularly in *G. barbadense*, which exhibits superior fiber strength. Therefore, this study analyzed the evolutionary relationships of TPX2 family members across four cultivated cotton species. Subsequently, it explored the differential expression patterns and functional differentiation characteristics among TPX2 family members in *G. barbadense*. These findings lay the foundation for a deeper exploration of the TPX2 family members’ mechanisms in the development of *G. barbadense* fibers.

## 2. Materials and Methods

### 2.1. Identification of TPX2 Family Members in G. barbadense

Amino acid sequences of TPX2 family members in *A*. *thaliana* were obtained from the *TAIR* database (https://www.arabidopsis.org/, accessed on 3 February 2025). Genome data for *G. barbadense* 3-79, *G. hirsutum* TM-1, *Gossypium raimondii*, and *Gossypium arboreum* were downloaded from the CottonGen database (https://www.cottongen.org/, accessed on 3 February 2025) using the latest publicly available genome assemblies and annotation releases. And TBtools (TBtools v1.128, CJ-chen Lab, South China Agricultural University, Guangzhou, Guangdong, China) was used for data formatting and integration, followed by redundancy removal and gene merging [29]. Candidate TPX2 (PF06886) family members from the four cotton genomes were initially identified from the Pfam database (http://pfam.xfam.org/, accessed on 3 February 2025) with a strict E-value cutoff of 1 × 10^−10^ and domain coverage ≥ 60% to ensure domain authenticity. And subsequently validated through bidirectional BLASTP searches in the NCBI database (http://www.ncbi.nlm.nih.gov/, accessed on 5 February 2025) with screening parameters set as E-value < 1 × 10^−10^ amino acid identity ≥ 30%, and query coverage ≥ 50%. The final number and sequence sets for TPX2 family members in each cotton genome were then determined. Molecular weight and isoelectric point analyses were conducted using the EXPASY platform (https://www.expasy.org/, accessed on 5 February 2025). Sequence integrity was assessed and corrected with the Softberry tool (http://softberry.com/, accessed on 8 February 2025)).

### 2.2. Construction of Phylogenetic Tree

Using the full-length protein sequence of TPX2 from the model plant *A*. *thaliana* as a reference, TPX2 family members were screened and identified in four cotton genomes. Subsequently, the obtained cotton TPX2 sequences were aligned with the *A*. *thaliana* reference sequence using the ClustalW algorithm in MEGA 11 [30]. A phylogenetic tree was constructed using the maximum likelihood (ML) method, with 1000 bootstrap replicates to ensure the reliability of the results. Bootstrap values were strictly used to delineate the same evolutionary branch. Visualized in iTOL (https://itol.embl.de/, accessed on 12 February 2025).

### 2.3. Analysis of Gene Structure, Conserved Domains, Chromosomal Localization, and Collinearity

The chromosomal localization information for the TPX2 genes was obtained from the cotton genome database. The gene structure, conserved motifs, and associated visualizations were generated using the AOGV module in TBtools. Chromosomal maps of TPX2 family members were created with TBtools. Collinearity analyses within and between genomes were performed using TBtools combined with MCScanX.

### 2.4. Cis-Element Analysis

The 2.0 kb upstream sequences from the ATG start codon of TPX2 genes were analyzed in PlantCARE (http://bioinformatics.psb.ugent.be/webtools/plantcare/html/, accessed on 13 February 2025) to identify cis-regulatory elements.

### 2.5. Gene Expression Analysis

Ovules at 0 day post anthesis (DPA) and mixed ovule and fiber samples at 5 DPA, as well as fibers collected at 10, 15, 20, 25, 30, and 35 DPA, were obtained from PimaS-7, 5719 and three *G. barbadense* accessions with high fiber strength and three accessions with low fiber strength. For samples from 10 to 35 DPA, fibers were rapidly dissected on ice and immediately frozen in liquid nitrogen. Specifically, we selected the GhUBQ7 as the internal reference for normalization. All qRT-PCR reactions were performed with three independent biological replicates and three technical replicates per biological sample to ensure data reliability. The relative expression levels were calculated using the method. Statistical significance was determined via ANOVA, with differences considered significant at *p* < 0.05. qRT-PCR primers with an amplicon size of approximately 250 bp were designed using Primer Premier 5 (Primer Premier 5.0, PREMIER Biosoft International, Palo Alto, CA, USA) (Table S1). All plant materials were planted in Aksu (Xinjiang Province, 4117007.12N, 8026050.68E) for consecutive years.

All RNA-seq raw data were obtained from the NCBI database (https://www.ncbi.nlm.nih.gov/geo/query/acc.cgi?acc=GSE178945, accessed on 23 February 2025). Quality control of raw reads was performed using FastQC software (FastQC v0.11.9, Babraham Bioinformatics, Cambridge, UK), and low-quality reads and adapter sequences were removed via Trimmomatic. Using the *G. barbadense* genome as the reference genome [31]. The quality-controlled clean reads were aligned to the reference genome of the corresponding cotton species using HISAT2 software (HISAT2 v2.2.1, Center for Computational Biology, Johns Hopkins University, Baltimore, MD, USA) with default parameters. The read counts aligned to each TPX2 gene were quantified using String Tie, and the expression levels were normalized to FPKM (Fragments Per Kilobase of transcript per Million mapped reads) values to eliminate the interference of gene length and sequencing depth. Subsequently, the normalized FPKM values were used for generating gene expression heatmaps via TBtools.

### 2.6. Virus-Induced Gene Silencing Analysis (VIGS)

Receptor materials, 5917, were cultivated in a controlled cotton growth chamber. Selected healthy seeds were germinated indoors, and VIGS primers were designed with the Primer Premier 5. A 300–380 bp fragment of the GbTPX2-35 was amplified from cDNA. The PCR products digested with SpeI were inserted into SpeI cut CLCrV-VIGS. Plasmids were extracted, and seamless ligation was used to insert target fragments into the linearized vector before transformation into Agrobacterium tumefaciens GV3101. After transformation, the positive Agrobacterium carrying the target fragment was activated and propagated in YEB medium (28 °C, 16–18 h, OD = 1.8–2.0). The bacterial suspension was centrifuged, and the pellet was resuspended in the prepared resuspension solution (10 mM MgCl, 10 mM MES, 200 μM acetosyringone) to an OD of 1.3. The Agrobacterium suspension was introduced into leaves by gently scratching the surface via a needle prick method and infiltrating the bacterial solution. Using wild-type seedlings as the control. Plants were watered regularly and maintained under a 16 h light cycle. Seedlings with fully expanded cotyledons and uniform growth were selected for subsequent experiments.

Plants with significantly down-regulated expression of the target gene after silencing were selected and continuously cultured until cotton fiber maturation. Cotton fiber samples were then collected for subsequent analysis. Each material no less than 15 g of fiber samples, with three biological replicates and three technical replicates. Fiber strength was determined using a high-volume instrument (HVI) in accordance with the international standards for cotton fiber quality testing.

## 3. Results

### 3.1. Identification of TPX2 Genes in Cotton

To identify TPX2 family members in cotton, nine characterized TPX2 sequences from A. thaliana containing the TPX2 (PF06886) domain were used to screen the protein databases of *G. barbadense*, *G. hirsutum*, the D-subgenome species *G. raimondii*, and the A-subgenome species *G. arboreum*. This search retrieved 152 sequences. After confirming the presence of the TPX2 domain and removing incomplete or redundant entries, 141 protein sequences were retained. Among these, 40, 49, 26, and 26 originated from *G. barbadense*, *G. hirsutum*, *G. arboreum*, and *G. raimondii*, respectively. In *G. barbadense*, TPX2 proteins ranged from 117 to 783 amino acids, with a mean length of 467.6 amino acids. Their isoelectric points spanned from 6.56 to 10.02, and only the isoelectric point (pI) of *GbTPX2-03* was less than 7. This result suggests that the GbTPX2 family is predominantly enriched in basic amino acid residues (Table S2).

### 3.2. Evolutionary Analysis of TPX2 Genes in Cotton

**Figure 1:**
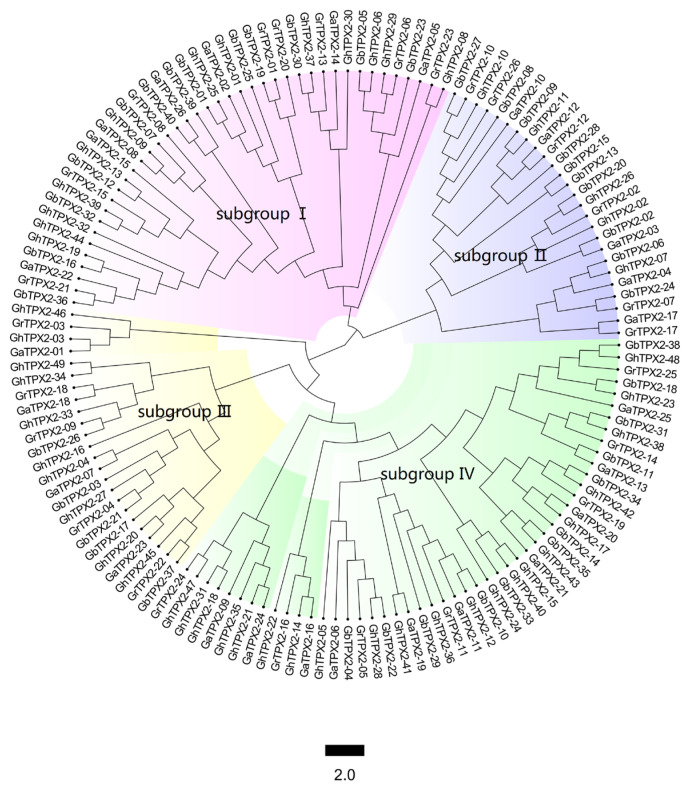
Phylogenetic tree of TPX2 proteins. The plant sequences were aligned using MEGA11, and a bootstrapped neighbor-joining tree was constructed. Four main snbgroups were identified (colored nodes).

To assess the evolutionary relationships of TPX2 genes in cotton, conserved amino acid sequences from the four species were analyzed. TPX2 sequences were aligned, and a phylogenetic tree was constructed using the neighbor-joining method (Figure 1). The clustering of multiple branches reflects conserved functions derived from a common ancestor. The resulting phylogeny divided the TPX2 protein sequences into four major subgroup, all 40 identified TPX2 family members from *G. barbadense* exhibit an uneven distribution across the four major subgroups of the phylogenetic tree. All identified members contained the TPX2 domain, although their functions were not identical. Subgroup I includes WDL7, subgroup II includes WDL5/6, subgroup III includes TPX2, and subgroup IV includes WVD\WDL2\3\4. Notably, each subgroup contains orthologous genes from four cotton species. This unique phylogenetic topology further suggests that there may be functional differentiation among different subgroups.

### 3.3. Collinearity Analysis and Chromosomal Localization of TPX2 Genes in Cotton

**Figure 2:**
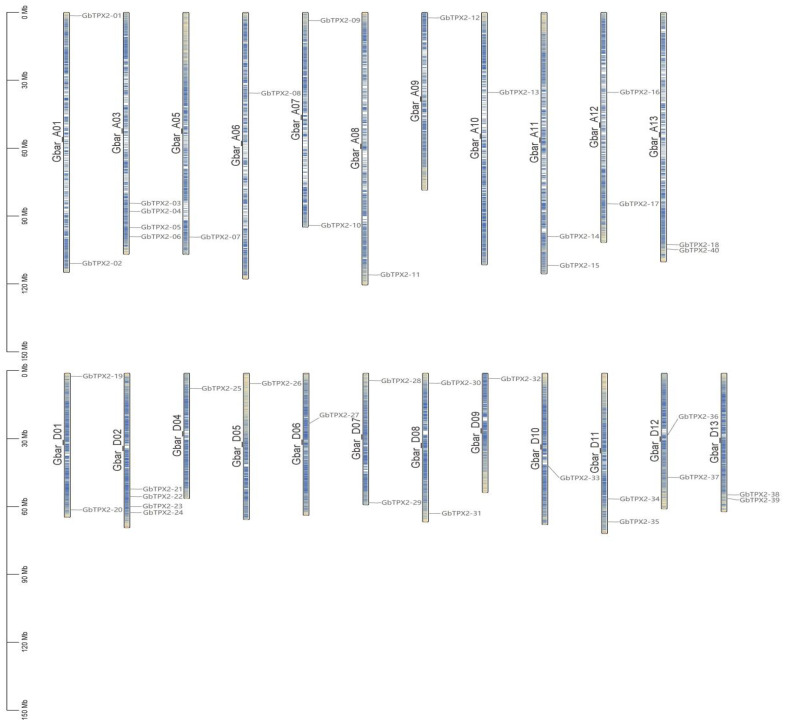
Chromosomal localization of TPX2 family members in *Gossypium barbadense*.

The collinearity between TPX2 family members in *G. barbadense*, *G. hirsutum*, *G. raimondii*, and *G. arboreum* was analyzed using MCScan software (Figure S1). The 40 TPX2 genes in *G. barbadense* were distributed across 23 chromosomes, excluding A02, A04, and D03 (Figure 2). Similarly, the 49 TPX2 genes in *G. hirsutum* spanned 23 chromosomes but were absent from A02, A04, and A08 (Figure S2A). The 26 TPX2 genes in *G. arboreum* were distributed across all 13 chromosomes (Figure S2B), whereas the 26 genes in *G. raimondii* were located on 13 chromosomes (Figure S2C). Overall, the TPX2 genes of the four cotton species were broadly dispersed—typically with one to two genes per chromosome—and were predominantly located toward distal chromosomal regions. However, several chromosomes contained clusters of 3–4 genes. For example, four genes were clustered on the long arm of chromosome D03 in *G. raimondii* and on chromosome A03 in *G. arboreum*. Similar clusters were present on chromosome A03 in *G. hirsutum* and *G. barbadense*. Notably, TPX2 genes were absent in chromosomes A02 and A04 in both tetraploids. These distribution patterns are consistent with the evolutionary relationships between diploid and tetraploid cotton species, suggesting gene loss or variation during polyploid evolution.

### 3.4. Analysis of Gene Structure, Motifs, and Cis-Acting Elements

**Figure 3:**
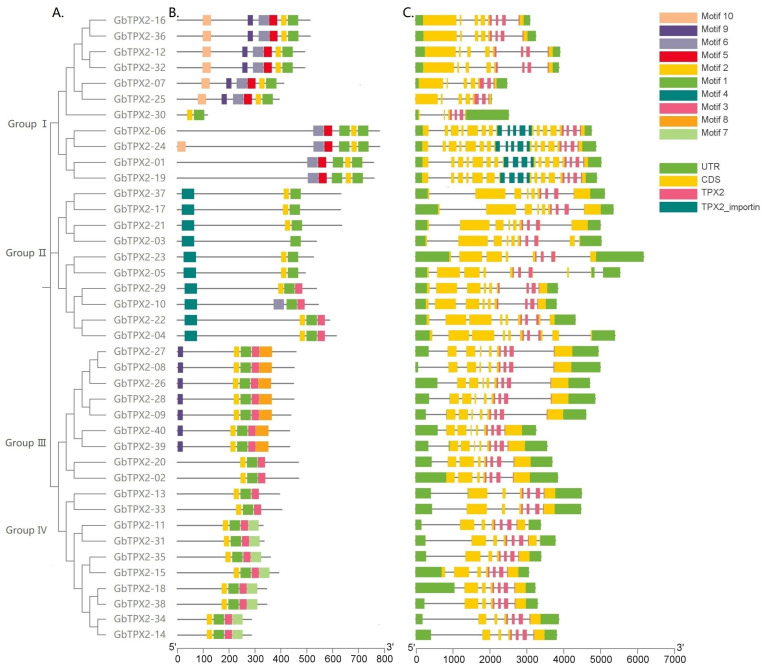
Phylogenetic relationship, conserved motifs, and structures of TPX2 family members in *G. barbadense.* An alignment of the TPX2 domain sequences was then obtained from the PFAM database. (**A**) A phylogenetic tree of the TPX2 gene family in *G. barbadense*. (**B**) Conserved motifs of the TPX2 gene family in *G. barbadense*. (**C**) The gene structure and functional domain distribution plot of the TPX2 family members in *G. barbadense*.

A conserved TPX2 domain (PF06886) was identified in all members of the *G. barbadense* TPX2 family. The conserved motifs were predicted using the MEME algorithm, and the distribution of these motifs was analyzed across all GbTPX2 proteins (Figure 3). A total of 10 distinct conserved motifs were detected, with motifs 1 and 2 being universally present, forming the core of the typical TPX2 domain. Notably, all members of Group III and Group IV stably contain motif 3, while this motif is completely absent in other subgroups, suggesting that it may be involved in lineage-specific functional specialization. In contrast, Groups I and II exhibit more variable motif compositions, with motifs 4 and 5 sporadically distributed, implying that they may play adaptive roles in specific developmental contexts.

Furthermore, some members of Group II (GbTPX2-37, GbTPX2-17) contain an additional conserved domain, TPX2_importin, which is associated with nuclear localization and interaction with importin-α, suggesting their potential involvement in regulating spindle assembly during cell division. The study also identified several truncated GbTPX2 proteins (GbTPX2-30, GbTPX2-06) that lack partial segments of the TPX2 domain or additional motifs. These structural variations may arise from intron insertion, exon deletion, or frameshift mutations during evolution, leading to partial loss of microtubule-binding activity or regulatory functions. In summary, the differences in motif composition and domain configuration among subgroups provide a structural basis for the functional diversification of the TPX2 family in *G*. *barbadense*, which may be closely related to their specific roles in processes such as fiber cell elongation and cell wall biosynthesis.

Based on the phylogenetic tree and the promoter cis-acting element distribution of 40 TPX2 family members in *G. barbadense*, these genes were clustered into four distinct groups, which exhibit a significant functional specialization in cis-element categories (Figure S3). A strong collinearity was observed between phylogenetic clades and cis-element profiles: Group I is enriched in light-responsive elements, Group II in gibberellin-responsive elements; Group III and Group IV in meristem-expression-related elements, with most of these elements predominantly concentrated in the proximal promoter region. Collectively, these findings not only elucidate the transcriptional regulatory basis underlying the functional specialization of the TPX2 family but also present critical cis-acting elements as candidates for functional validation in cotton fiber development. In cotton fiber development, there may be new targets for improving fiber quality through transcriptional regulation.

### 3.5. Expression Pattern Analysis

**Figure 4:**
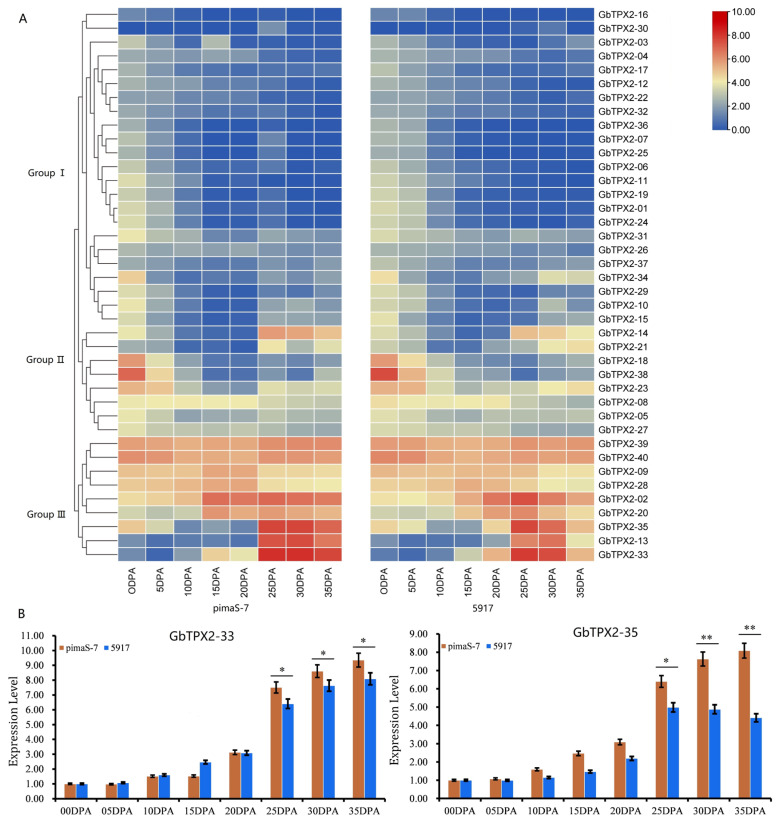
Expression analysis of *G. barbadense* TPX2 family members in different tissues. (**A**) Expression levels of 40 GbTPX2 genes were retrieved from transcriptome datasets. (**B**) Expression levels of five GbTPX2 genes were quantified via quantitative reverse transcription polymerase chain reaction (qRT-PCR) in cotton fiber tissues. Error bars represent ± SD of three or more biological replicates. Level of significance: * *p* < 0.05; ** *p* < 0.01.

To further characterize the family, expression levels of 40 TPX2 genes were retrieved from transcriptome datasets (Figure 4A). In *G. barbadense*, TPX2 genes were classified into three groups. Group I, with 23 genes, demonstrated a consistently low expression during the fiber development from 0 to 35 DPA. Group II, which contained eight genes, exhibited a slightly elevated expression during fiber initiation relative to other stages. Group III consisted of nine genes; four genes are persistently expressed throughout the four stages of fiber development with no significant differences in expression levels among these stages. The remaining five genes show distinct differential expression patterns across the four fiber developmental stages, with particularly prominent changes during the fiber elongation and secondary cell wall biosynthesis stages. The five genes in group III that are highly expressed during cotton fiber’s secondary cell wall development were selected for expression-level validation. This validation was performed using cotton fibers of PimaS-7 and 5917 collected at 0–35 days post-anthesis (DPA) (Figure 4B and Figure S4). Overall, three genes showed significant differences at multiple time points during the secondary wall thickening stage of the samples. The expression trends of these genes were consistent with the results observed in the transcriptome data.

The three genes that belong to Group III and were highly expressed during secondary cell wall development were further validated (Figure S4). Three *G. barbadense* accessions with high fiber strength and three with low fiber strength were selected, and a qRT-PCR was conducted on fiber tissues collected from 0 to 30 DPA. During the primary formation of the cell wall (0–15 DPA), the expression of all three genes remained low and consistent, with patterns observed in the parental lines and no significant differences between materials that differ in fiber strength. At 20 DPA, the expression in the high-fiber-strength accessions began to increase more distinctly than in the low-fiber-strength accessions. By 25 and 30 DPA, the transcript levels exhibited a continued upward trend across all materials, with a stronger increase at 30 DPA. In high-fiber-strength accessions, the upregulation of the three genes accelerated at 25 DPA and peaked at 30 DPA. The GbTPX2-33, GbTPX2-35, and GbTPX2-13 genes clustered in Group III exhibited significantly high expression levels specifically during the secondary wall development stage, further suggesting the potential involvement of these microtubule-associated proteins in regulating the formation of fiber strength traits.

Furthermore, this result confirms that a distinct divergence in the expression pattern exists among different members of the same GbTPX2 gene family; such a divergence in expression patterns indicates a significant correlation with the functional differentiation of the genes.

### 3.6. VIGS

**Figure 5:**
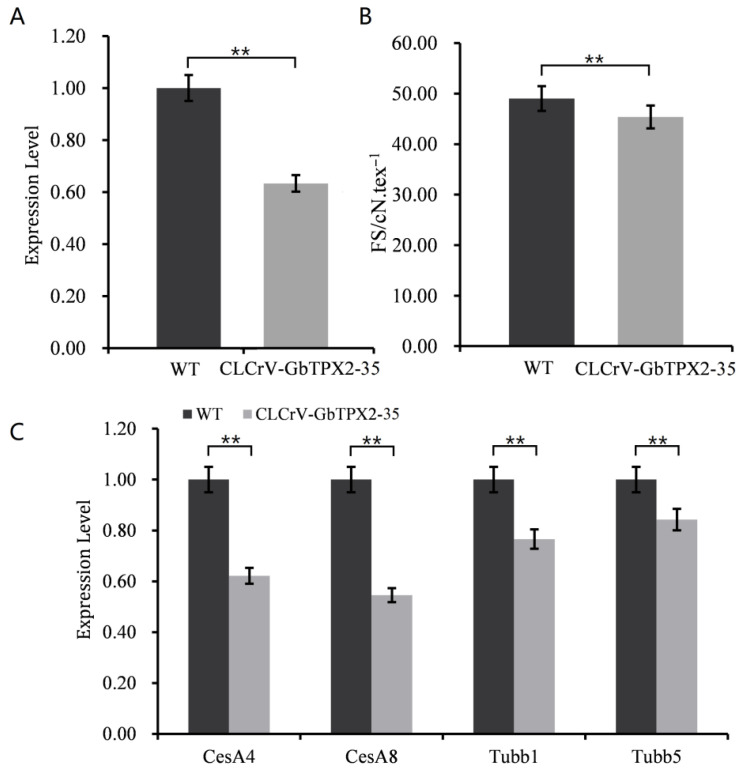
GbTPX2-35 positively regulates cotton fiber strength during the secondary cell wall thickening stage of fiber development. (**A**) The relative expression level of GbTPX2-35 in the control and CLCrV-GbTPX2-35. (**B**) The fiber strength (FS, cN/tex-1) of the control and CLCrV-GbTPX2-35. (**C**) The transcript abundances of *CESA4*, *CESA8*, *TUBB1*, and *TUBB5* were significantly reduced upon GbTPX2-35 silencing, as determined by the RT-qPCR analysis. Error bars represent ± SD of three or more biological replicates. Level of significance: ** *p* < 0.01.

*G. barbadense* PimaS-7 was used as the experimental material for virus-induced GbTPX2-35 gene silencing. A qRT-PCR analysis revealed that the expression level of GbTPX2-35 in VIGS-silenced plants was significantly lower than that in the control plant (Figure 5A). After the cotton had naturally matured, fiber samples were collected for strength determination (Figure 5B). These phenotypic results further demonstrate that reducing the expression of GbTPX2-35 directly affects the development of cotton fiber strength traits.

Silencing GbTPX2-35 significantly reduced *CesA4* and *CesA8* transcript levels compared with the control plant, indicating that the suppression of the microtubule-associated protein downregulates these cellulose synthase genes (Figure 5C). This finding exhibits a similar trend to the results reported by Cao and Huang et al. [32,33]. Transcript levels of two tubulin genes were also reduced following GbTPX2-35 silencing, with *Tubb1* exhibiting the most pronounced decrease (Figure 5C). This finding demonstrates that the reduced expression of the microtubule-associated protein GbTPX2-35 may also impact the transcriptional levels of microtubule genes [34,35]. These findings indicate that regulating GbTPX2-35 can partially suppress the expression of microtubule-associated genes tubb1 and tubb5, as well as cellulose synthase genes, further suggesting that there may be a positive regulatory mechanism among the three during cellulose formation and rapid deposition stages.

## 4. Discussion

The TPX2 gene family contains two core conserved domains: the TPX2 (XKLp2) conserved motif, which is responsible for mediating microtubule binding, and the TPX2–importin domain involved in nuclear transport.

In this study, 141 TPX2 genes were clustered into four main clades based on phylogenetic analysis, which is consistent with the findings reported [27]. However, this result is different from previous studies that classified plant TPX2 genes into six clades [23]. This discrepancy may be attributed to the fact that only one conserved motif of the canonical TPX2 family, the TPX2 (XKLp2) motif (PF06886), was considered when screening the functional domains of TPX2 genes in *G. barbadense*. In the present study, a total of 40 GbTPX2 genes were identified in *G. barbadense*. In contrast to the 49 GhTPX2 genes previously reported in *G. hirsutum*, several GbTPX2 homologs have been lost during the evolutionary process. This pattern is consistent with the established notion that allotetraploids exhibit a relatively high frequency of gene loss during evolution, suggesting a potential association with the domestication-driven improvement of fiber quality in *G. barbadense*.

Prior gene silencing assays on *G. hirsutum* have demonstrated that silencing GhWDL3 impairs fiber cell elongation [27]. Consistently, VIGS-mediated silencing of GbTPX2-35 in *G. barbadense* significantly reduced fiber strength, accompanied by downregulated expression of *CesA4* and *CesA8.* Previous studies have demonstrated that cellulose synthases (*CesA*) are assembled in the Golgi apparatus and then transported to the plasma membrane (PM) [36]. At the plasma membrane, *CesA* catalyzes cellulose synthesis and mediates the assembly of microfibrils in the secondary cell wall (SCW) [37,38]. The transcription factor *GhMYB7* positively regulates the expression of *GhCesA* genes by binding to specific cis-acting elements in their promoter regions, thereby promoting secondary cell wall cellulose deposition in cotton fibers [33]. These cellulose synthase genes function predominantly during the second phase of fiber development, when cellulose synthesis and deposition accelerate [33,39]. In *G. hirsutum*, *CesA* and *CesA8* exhibit significantly higher expression levels during secondary wall thickening at 20–25 DPA, relative to wild cotton species, and their enhanced activity through domestication has improved their fiber quality [40]. Other studies have provided detailed explanations for the above phenomenon.

In vivo observations have revealed that the microtubule array not only guides the transport of cellulose synthase complexes, but also regulates their docking and secretion at the plasma membrane, with its orientation determining the direction of cellulose synthesis [41,42]. The rate, deposition orientation and uniformity of cellulose synthesis influence the arrangement of fiber microfibrils, ultimately determining the crystalline structure of cellulose and the mechanical strength of the cell wall [37,38,43]. The orientation of cellulose microfibrils dictates the crystalline structure of cellulose, thereby affecting the formation of fiber strength traits [44].

*Tubb1* and *Tubb5* are the core components of microtubule assembly [45,46]. In this study, their expression of both genes decreased overall following GbTPX2-35 silencing, despite individual fluctuations. Cellulose microfibrils align perpendicular to the cell wall, while cortical microtubules align parallel to it [47]. Microtubule depolymerization in *A*. *thaliana* significantly disturbs the directional growth of root hairs, a finding corroborated by multiple studies [48,49]. Early investigations since 1963 have confirmed that microtubules govern cellulose arrangement in plant root cells, and microscopic observations have directly visualized the movement of cellulose synthases along microtubule tracks [25,41]. In studies on the csi1/pom2 mutant, the movement rate of cellulose synthases was reduced by approximately two-thirds compared with the wild type, while a weaker *CESA* signal was detected on microtubules [50,51]. Microtubule arrays in the korrigan1 and procuste1/cesa6 mutants were observed to be significantly disorganized [7,52]. Taken together, these findings indicate that microtubule genes that regulate the microtubule cytoskeleton structure, microtubule-associated genes involved in modulating microtubule function, and cellulose synthases interact with each other to dynamically maintain the movement rate and localization of *CESAs* on microtubules. The disruption of any component of this regulatory network perturbs the dynamic balance [23,53]. The gene expression data in this study only preliminarily confirms the existence of real signal transduction processes in this regulatory network, providing important research ideas and directions for exploring the regulatory mechanism of microtubule-mediated cellulose polymerase complexes in the formation of fiber strength. However, to elucidate the specific signal mechanisms behind this regulation, further research through in vitro biochemical experiments and stable transgenic lines is still required.

## 5. Conclusions

This study identified and systematically characterized the TPX2 gene family in *G. barbadense*, and revealed that GbTPX2-35 is significantly upregulated during fiber secondary cell wall thickening. VIGS-mediated silencing of GbTPX2-35 downregulated cellulose synthase and tubulin gene expression, and markedly decreased mature fiber strength. We preliminarily validated a regulatory pathway wherein GbTPX2-35 modulates fiber strength by coordinating cellulose biosynthesis and microtubule cytoskeleton dynamics, highlighting the synergistic interplay between microtubule-associated genes and cellulose synthesis during fiber development. This work provides a valuable candidate gene and theoretical support for high-strength cotton fiber molecular breeding.

## Data Availability

All datasets generated during the current study are included in the main text or available in the online Supplementary Materials.

## Supplementary Materials

The following supporting information can be downloaded at: https://www.mdpi.com/article/10.3390/genes17040395/s1. Figure S1: Collinearity Analysis of Four Cotton Species; Figure S2: Chromosomal localization of TPX2 Family members in *Gossypium hirsutum*, *Gossypium raimondii*, *Gossypium arboretum*; Figure S3: Phylogenetic tree and promoter cis-acting element distribution of 40 TPX2 family members in *G. barbadense*.; Figure S4. Expression levels of GbTPX2 genes quantified by qRT-PCR in cotton fiber tissues. Figure S5. Expression analysis of three target genes during fiber development (0–30 DPA) via qRT-PCR. Three high-fiber-strength and three low-fiber-strength *G. barbadense* accessions were used as plant materials. Table S1: All primer sequences of VIGS and qRT-PCR; Table S2: The TPX2 family members in the databases of *G. barbadense*.
