# Supplementary material for: Functional Analysis of a Cotton TPX2-like Gene, GbTPX2-35, in Regulating Fiber Cell Development and Strength in Gossypium barbadense
Source: Genes (Basel). 2026 Mar 30;17(4):395. doi: 10.3390/genes17040395 (PMC13116628; doi:10.3390/genes17040395)
Supplement: Supplementary file 1 [file genes-17-00395-s001.zip › Supplementary Figures.pdf]

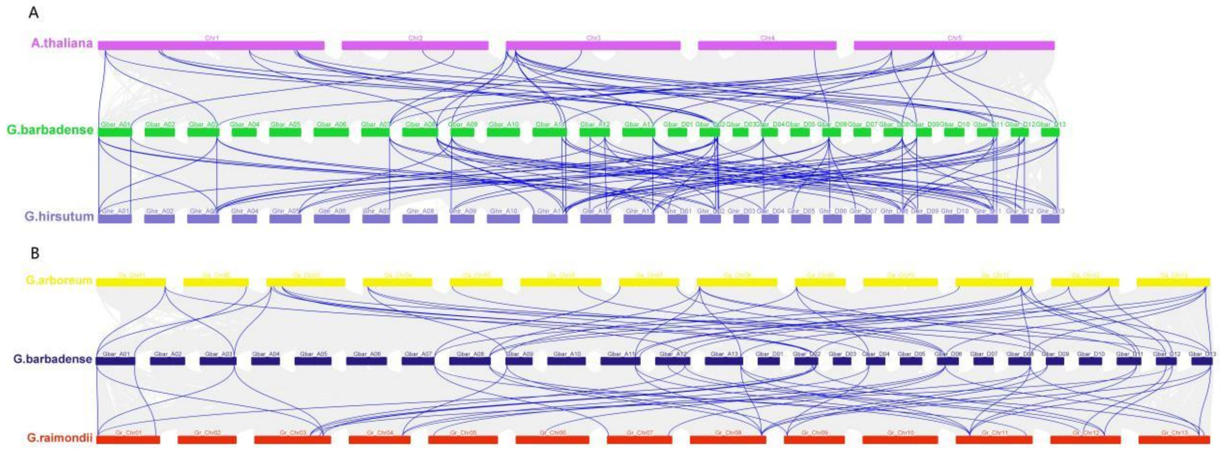

Figure S1. Collinearity Analysis of Four Cotton Species.

A.

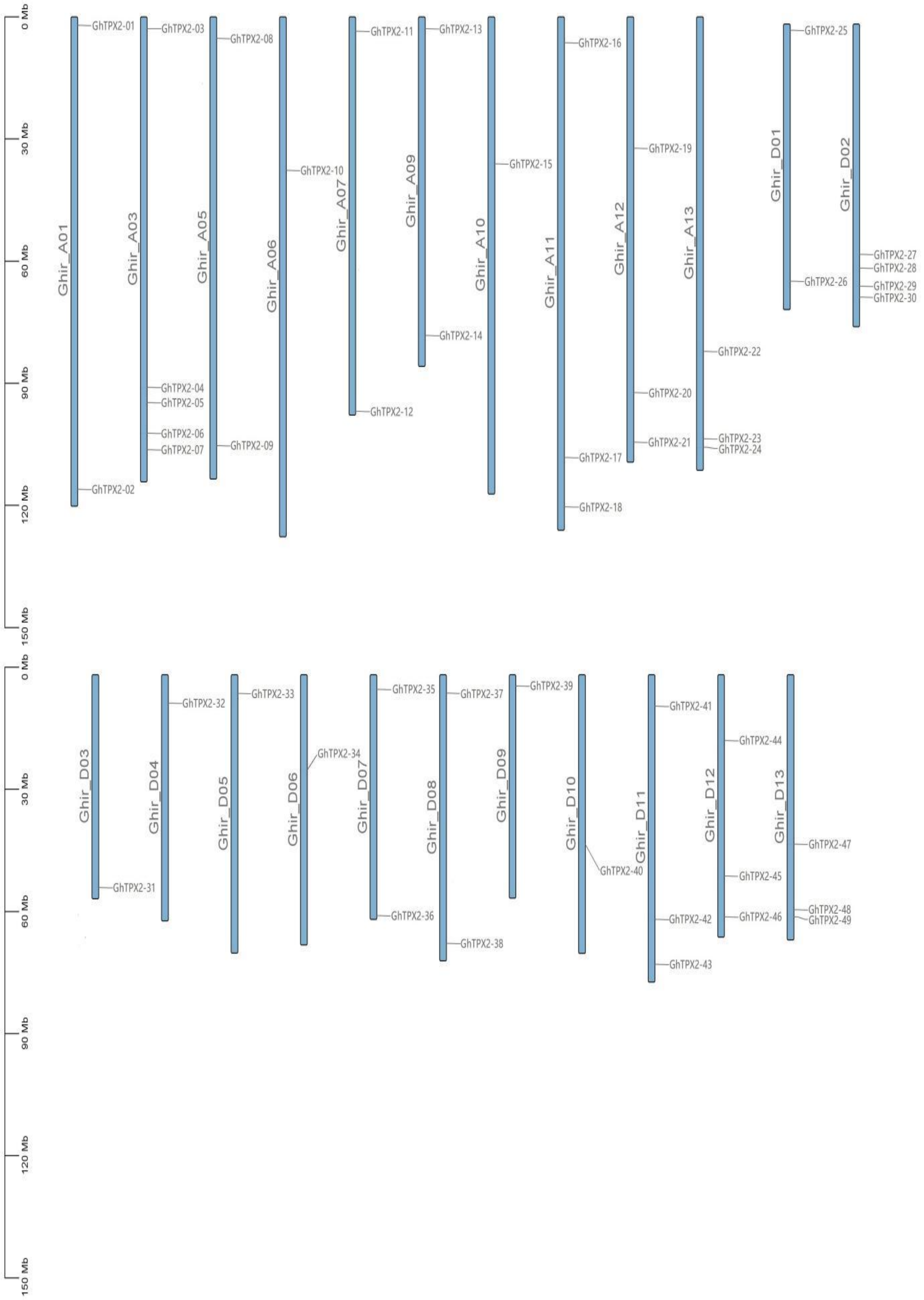

0 Mb
30 Mb
60 Mb
90 Mb
120 Mb
150 Mb
Ghir_A01
Ghir_A03
Ghir_A05
Ghir_A06
Ghir_A07
Ghir_A09
Ghir_A10
Ghir_A11
Ghir_A12
Ghir_A13
Ghir_D01
Ghir_D02
GhTPX2-01
GhTPX2-02
GhTPX2-03
GhTPX2-04
GhTPX2-05
GhTPX2-06
GhTPX2-07
GhTPX2-08
GhTPX2-09
GhTPX2-10
GhTPX2-11
GhTPX2-12
GhTPX2-13
GhTPX2-14
GhTPX2-15
GhTPX2-16
GhTPX2-17
GhTPX2-18
GhTPX2-19
GhTPX2-20
GhTPX2-21
GhTPX2-22
GhTPX2-23
GhTPX2-24
GhTPX2-25
GhTPX2-26
GhTPX2-27
GhTPX2-28
GhTPX2-29
GhTPX2-30
Ghir_D03
Ghir_D04
Ghir_D05
Ghir_D06
Ghir_D07
Ghir_D08
Ghir_D09
Ghir_D10
Ghir_D11
Ghir_D12
Ghir_D13
GhTPX2-31
GhTPX2-32
GhTPX2-33
GhTPX2-34
GhTPX2-35
GhTPX2-36
GhTPX2-37
GhTPX2-38
GhTPX2-39
GhTPX2-40
GhTPX2-41
GhTPX2-42
GhTPX2-43
GhTPX2-44
GhTPX2-45
GhTPX2-46
GhTPX2-47
GhTPX2-48
GhTPX2-49

B.

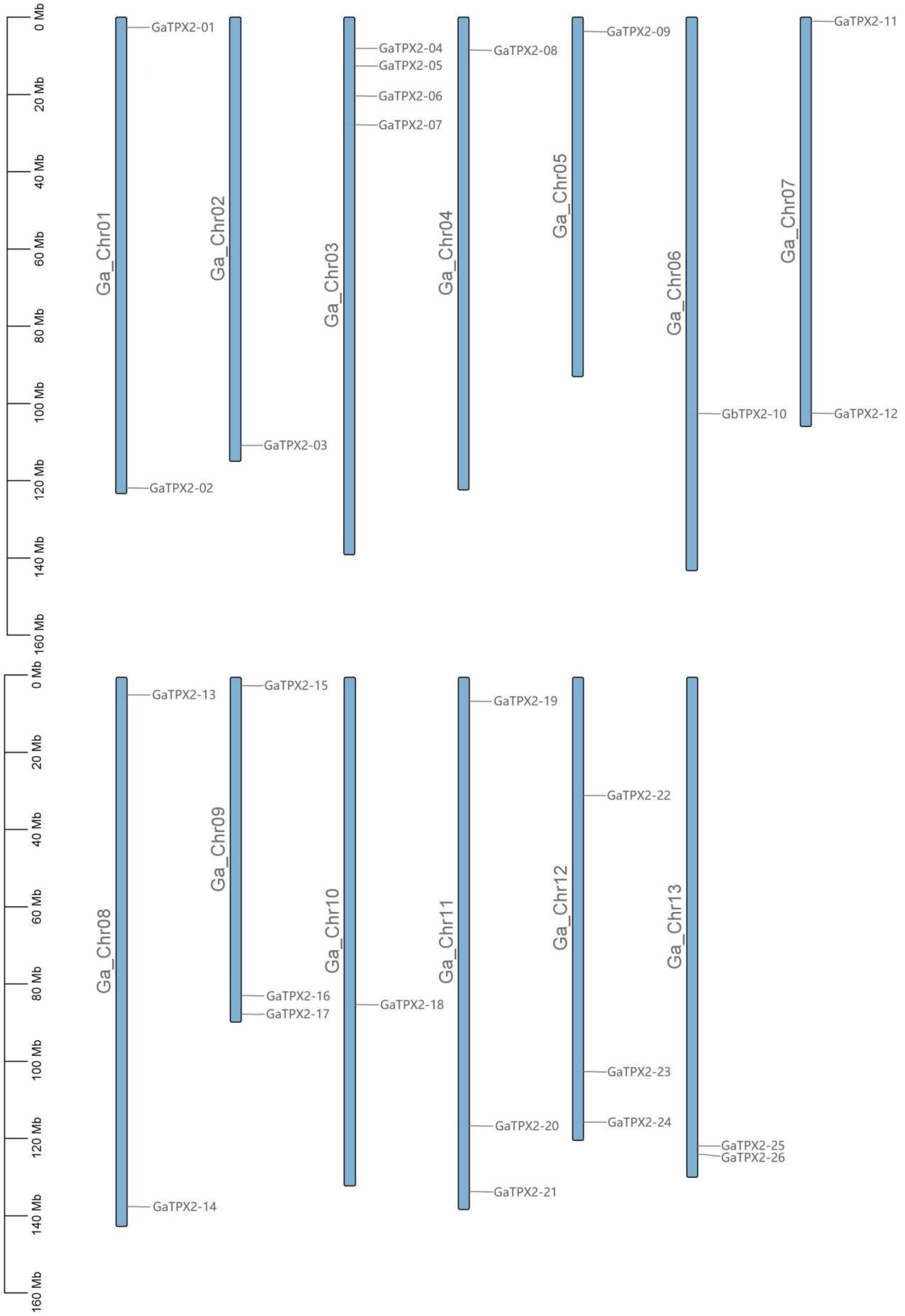
0 Mb
20 Mb
40 Mb
60 Mb
80 Mb
100 Mb
120 Mb
140 Mb
160 Mb
Ga_Chr01
GaTPX2-01
GaTPX2-02
Ga_Chr02
GaTPX2-03
Ga_Chr03
GaTPX2-04
GaTPX2-05
GaTPX2-06
GaTPX2-07
Ga_Chr04
GaTPX2-08
Ga_Chr05
GaTPX2-09
Ga_Chr06
GbTPX2-10
Ga_Chr07
GaTPX2-11
GaTPX2-12
Ga_Chr08
GaTPX2-13
GaTPX2-14
Ga_Chr09
GaTPX2-15
GaTPX2-16
GaTPX2-17
Ga_Chr10
GaTPX2-18
Ga_Chr11
GaTPX2-19
GaTPX2-20
GaTPX2-21
Ga_Chr12
GaTPX2-22
GaTPX2-23
GaTPX2-24
Ga_Chr13
GaTPX2-25
GaTPX2-26

C.

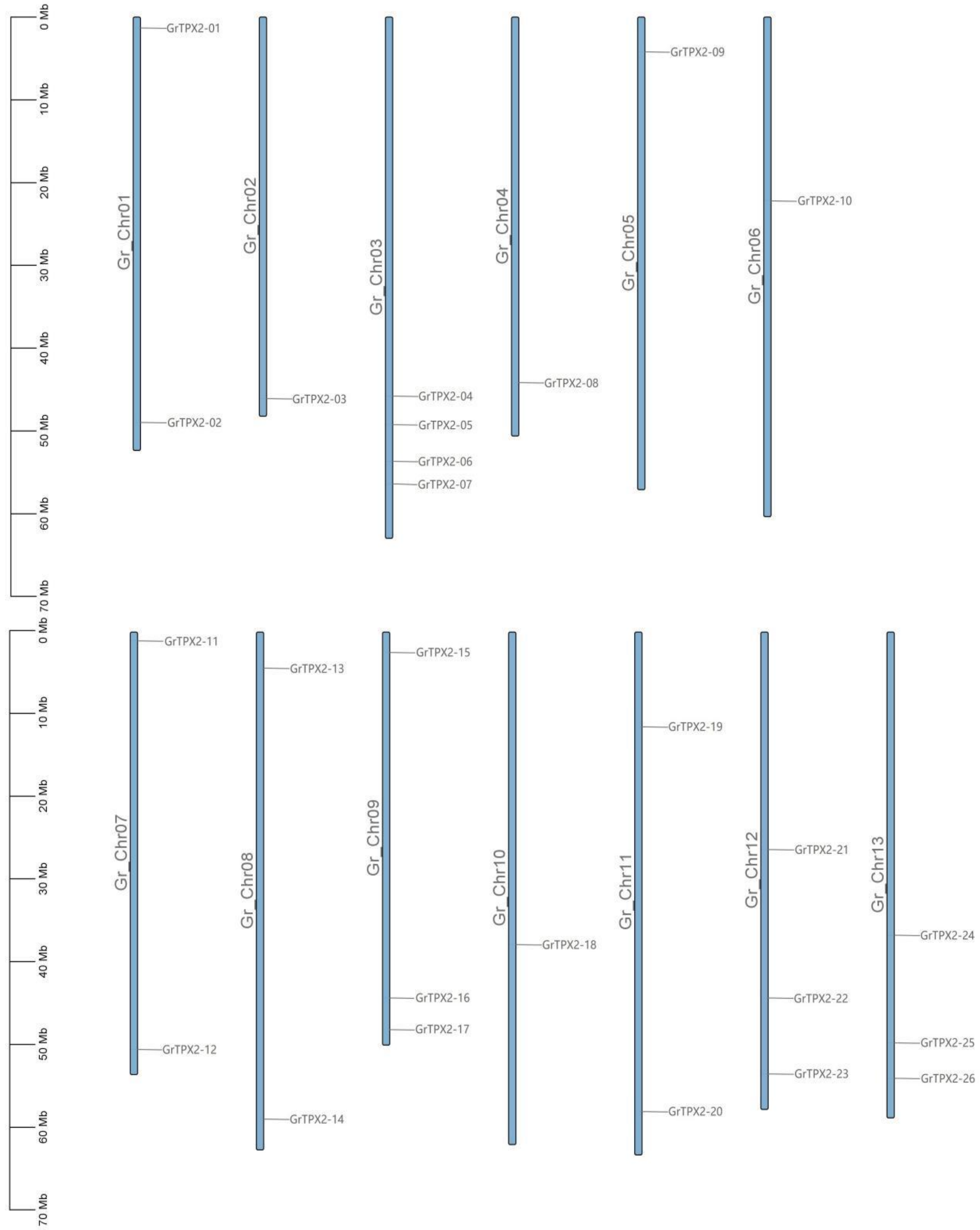

FigureS2. Chromosomal localization of TPX2 family members in *Gossypium hirsutum*、*Gossypium raimondii*、*Gossypium arboretum*.

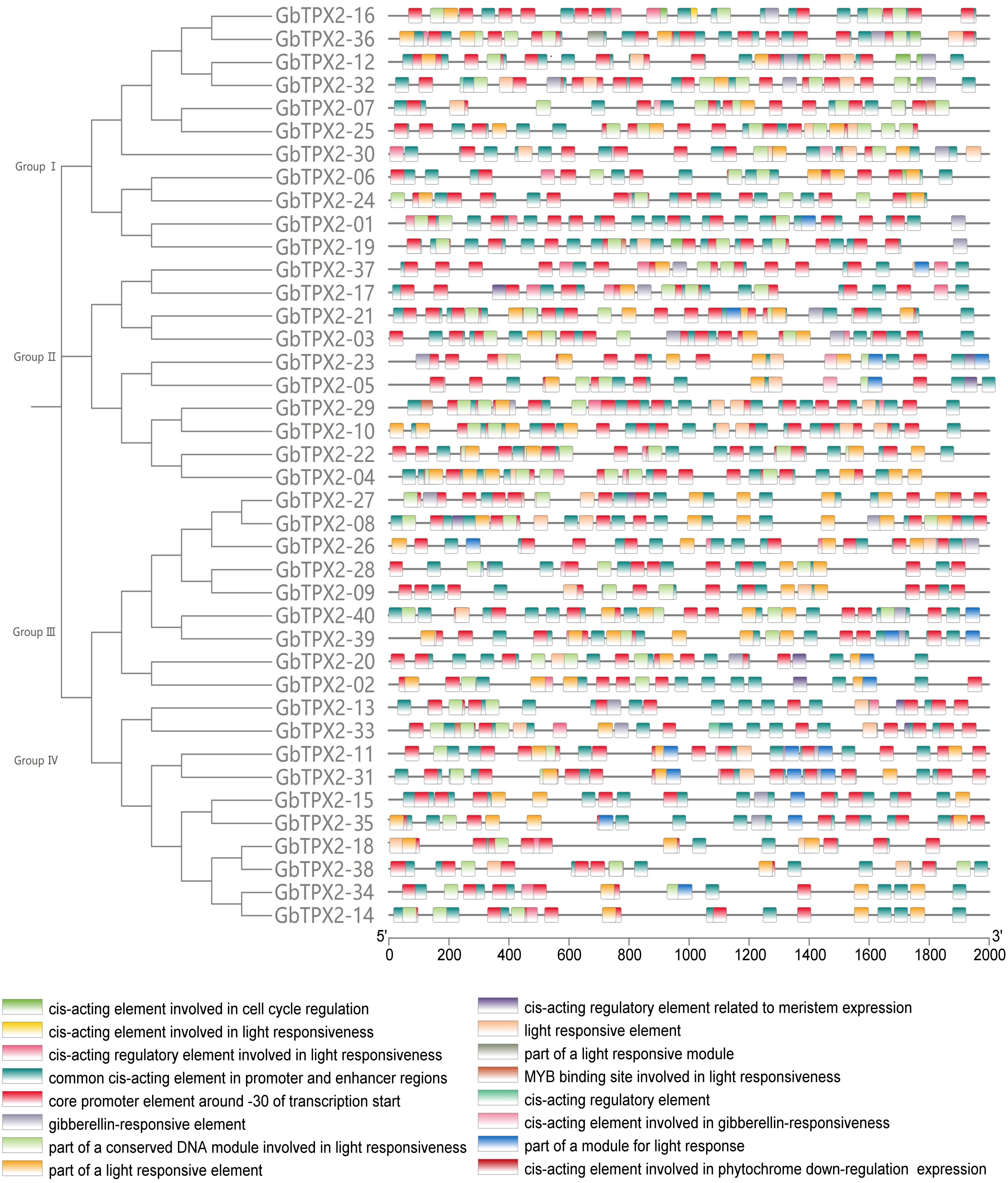

Figure S3. Phylogenetic tree and promoter cis-acting element distribution of 40 TPX2 family members in *G. barbadense.*

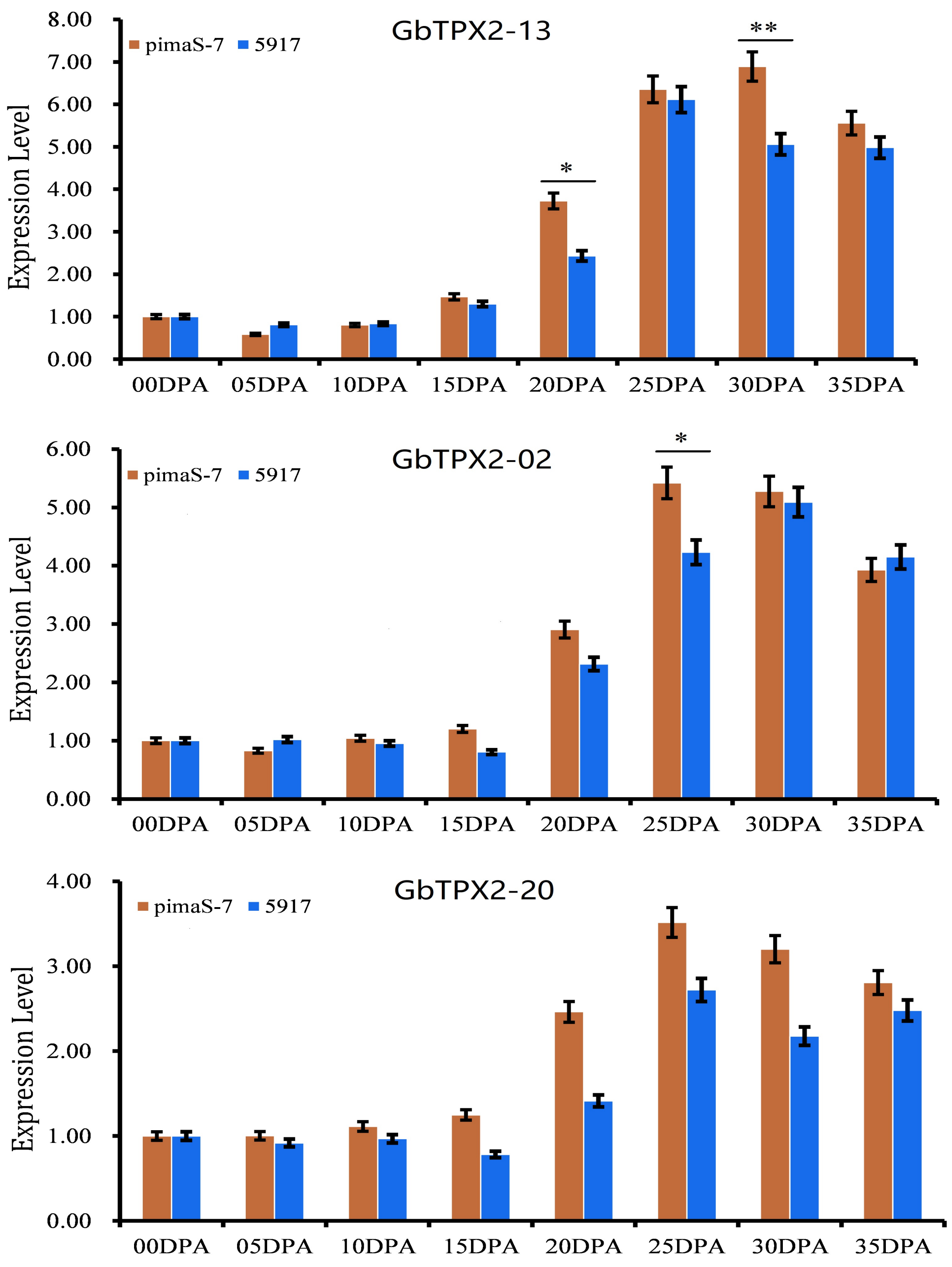

Figure S4. Expression levels of GbTPX2 genes was quantified by quantitative reverse-transcription polymerase chain reaction (qRT-PCR) in cotton fiber tissues. Error bars represent ± SD of three or more biological r eplicates. Level of significance: *P < 0.05; **P < 0.01.

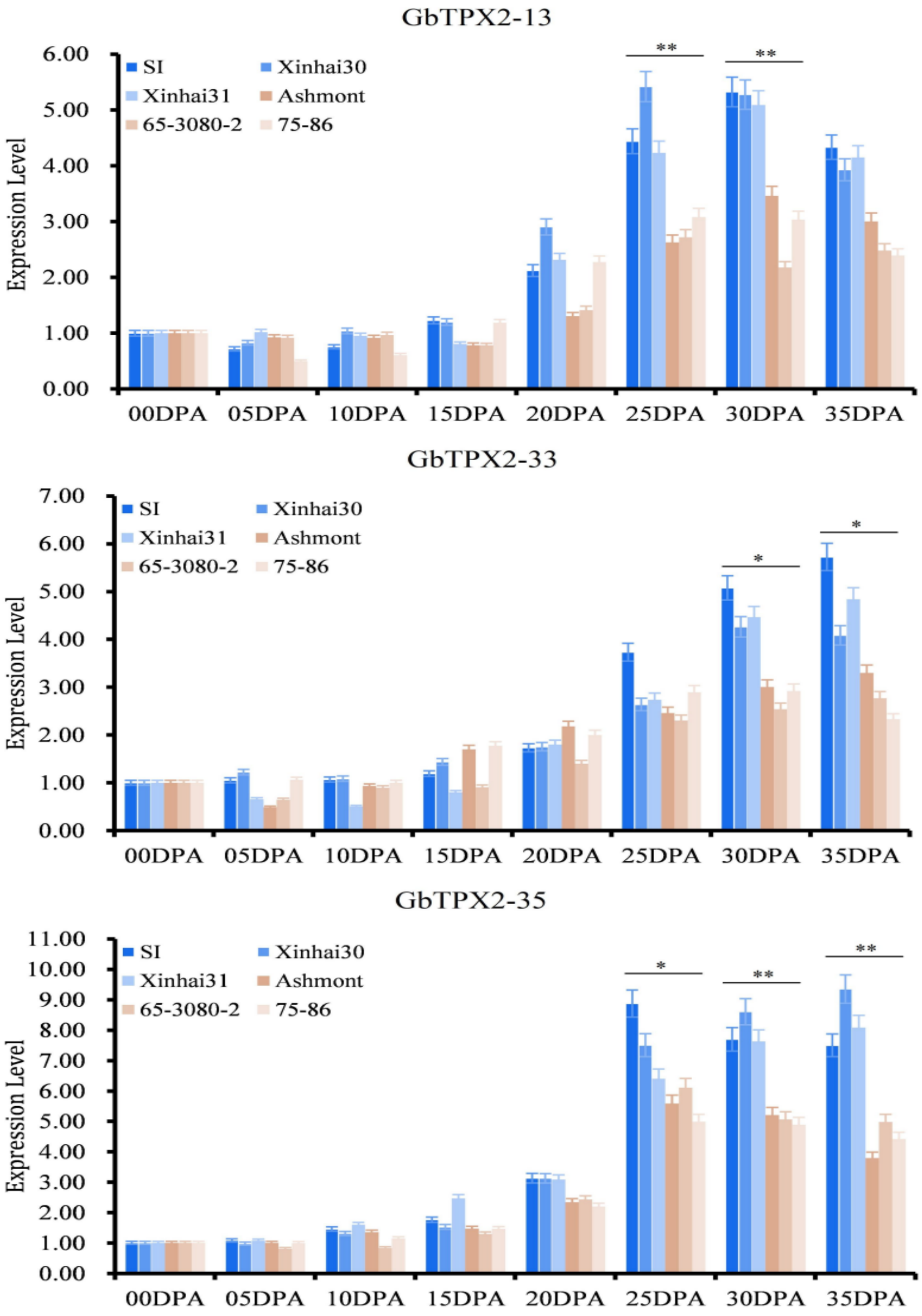

Figure S5. Expression analysis of three target genes during fiber development (0 – 30 DPA) via qRT-PCR. Three high-fiber-strength and three low-fiber-strength *G. barbadense* accessions were used as plant materials. Error bars represent ± SD of three or more biological replicates. Level of significance: *P < 0.05; **P < 0.01.
